# The Effects of Educational Campaigns and Smoking Bans in Public Places on Smokers' Intention to Quit Smoking: Findings from 17 Cities in China

**DOI:** 10.1155/2015/853418

**Published:** 2015-05-04

**Authors:** Biao Luo, Liang Wan, Liang Liang, Tieshan Li

**Affiliations:** ^1^Centre for Behavioural and Cognition Research, University of Science and Technology of China, Hefei, Anhui 230026, China; ^2^John Molson School of Business, Concordia University, Montreal QC, Canada H3G 1M8

## Abstract

Despite the perceived success of educational campaigns and smoking bans in public places in China, the actual effects have not been investigated. This study examines the effects of the two policies by major characteristics of smokers and whether the affected smokers have intention to quit smoking. A cross-sectional survey was conducted in 17 cities in China and 16,616 participants were selected using multistage stratified sampling. Logistic regression models were used to examine the effects of educational campaigns and smoking bans in public places on their intention to quit smoking. Results show that the Chinese government should try every means to build its tobacco control publicity and implement various forms of public educational campaigns to enhance smokers' knowledge of the health consequences of smoking. In addition, China should emphasize the enforcement of the existing smoking prohibitions and regulations by implementing local tobacco control legislation and total prohibitions in all public places and workplaces.

## 1. Introduction

Smoking is one of the leading preventable causes of death and diseases in the world. According to the World Health Organization (WHO), nearly six million people die annually from cigarette smoking [1]. China is the largest cigarette production and consumption country in the world, accounting for 38% of the world's cigarette production and sales. At present, approximately 320 million, nearly one-third of the world's smokers, are in China, and an estimated 738 million people are exposed to secondhand smoke [2, 3]. Approximately one million people die of tobacco induced diseases in China each year [4], a number that is projected to reach two million by the year 2020 [5]. If current smoking rate continues, according to the WHO, 100 million people in China under the age of 30 will die from tobacco use [6]. Thus, the tremendous pressure from tobacco induced diseases and death makes tobacco control (TC) an urgent concern in China. In addition, China plays a critical role in global TC efforts given the size of Chinese smokers.

The WHO Framework Convention on Tobacco Control (FCTC) has emphasized the importance of combining tobacco demand reduction with tobacco supply restrictions [7]. TC policies have been successfully carried out in the Western countries, and the lessons from these countries were helpful in drafting TC policies in developing countries such as China [8]. Given the unprecedented challenges and motivation on TC by Chinese government, this study focuses on examining the effects of public educational campaigns and smoking bans in public places in China.

### 1.1. Related Tobacco Control Policies in China

Smoking bans are public policies, including criminal laws and occupational safety and health regulations that prohibit tobacco smoking in workplaces and other public spaces. Educational campaigns are those utilizing integrated components to deliver messages (e.g., public service announcements, media literacy training, and classroom discussion), integrating the campaign into other tobacco education, counseling, and cessation. Smoking bans in public places have been ignored in China. China signed the FCTC in October 2003 and enforced the policy in 2006. In March 2011, the Chinese Ministry of Health (MOH) announced a new regulation of smoking bans in public places [9]. More than two years later, in December 2013, the Chinese government issued a notice, which was considered as the most strict regulation in China, implementing the smoking bans in public places in all geographical areas [10]. However, by far only 13 cities, including Beijing, Shanghai, Hangzhou, and Guangzhou, have adopted the legislation that enforces the ban of smoking in public places [11].

The educational campaigns on TC by health warning labels and antitobacco mass media campaigns were not promising either. Only text warnings have been used in the cigarette package without any pictorials. Detailed information about tar, nicotine, and carbon monoxide on the cigarette packages may mislead the public that a particular tobacco product is less harmful.

### 1.2. The Effects of Educational Campaigns and Smoking Bans in Public Places

Numerous theoretical studies and practices found that educational campaigns were effective in reducing tobacco use. Durkin et al. revealed that educational campaigns could promote quitting among adults and reduce adult smoking rates [12]. The recently released Surgeon General's report concluded that adequately funded antitobacco media campaigns could reduce tobacco use among the youth [13]. Similarly, the CDC's Best Practices for Comprehensive Tobacco Control Programs showed that public educational campaigns were an integral part of efforts to prevent the initiation of tobacco use and to encourage tobacco cessation [14]. Clearly, substantial evidence pointed to the effectiveness of educational campaigns in reducing the number of the youth who start smoking, increasing the number of smokers who quit, and making the tobacco industry marketing less effective, thereby saving lives and health care dollars [15, 16].

Smoking bans in public places have also been found effective in reducing tobacco consumption among smokers and reducing exposure to secondhand smoke [17]. These bans also contributed to the prevention of smoking uptake among children and young people by reshaping the perceived social acceptability of smoking [18]. The effectiveness of smoke-free policies was evident in numerous studies and countries [17, 19–21]. Moreover, in North America, Western Europe, and Australia, many of the TC efforts have focused on clean indoor-air laws, and the experiences of those regions are instructive to other countries which started working on TC [22–24].

### 1.3. This Study

Earlier studies on the effects of smoking bans and educational campaigns mainly focused on the influence of these policies on smoking behavior such as reducing smoking and choosing low-tar cigarette [25]. Some studies explored the difference in the scope of policy implementation (e.g., hospitality industry versus all workplaces) and difference in the effects of comprehensive and partial TC policies [26–28]. Other studies investigated the unintended consequences of TC policies, such as perceived stigmatization of smokers, trends in socioeconomic inequalities in smoking initiation, smoking prevalence, smoking consumption, and smoking cessation, and the impact of smoke-free legislation on voluntary home smoking bans [29–31].

In this study, we explore the TC policies in China by examining the role of individual-level factors in the effects of TC policies. Considering that most of the past studies were conducted in high-income countries, evaluation of the increased adoption of TC policies must be reviewed in low- and middle-income countries to examine the possible difference [32]. To the best of our knowledge, no studies have been done on individual-level factors associated with the effects of TC policies in China. Feng et al. used a sample of adult smokers in six Chinese cities to identify individual-level factors associated with intention to quit smoking [33]. However, they did not examine the effects of the two TC policies. Moreover, other earlier studies could not provide a comprehensive examination due to the lack of large-scale survey data [33–35]. In addition, the present study included children (household member aged 15 to 18), which is also a further step from the literature. Our study is the first one to completely examine the effects of TC policies on quitting smoke and the role of individual demographic characteristics in TC policies.

## 2. Materials and Methods

This study aims to explore, from a large-scale representative sample data, the relation between the individual-level factors of smokers and the effects of educational campaigns and smoking bans in public places and whether these two policies can drive smokers to quit smoking.

### 2.1. Study Design and Sampling

A cross-sectional survey was conducted from October 2010 to January 2011 in 17 cities in Anhui Province. Anhui lies in China's most economically developed regions (Eastern region), while economically it belongs to China's central economic zone. The primary sampling units were households and one household member aged 15 to 70 was randomly selected for the interview. The other inclusion criteria were as follows: (i) being ethnically Chinese and (ii) current smokers, either occasional or daily. The sample size was determined with the objective to ensure high statistical reliability and representativeness; the 95% CI should not exceed 5% of a key indicator estimate. From those criteria, the sample size was determined to be 24,000 and the sampling error was 0.63%, and a total of 16,616 valid questionnaires were collected (response rate of 69.2%). Of the 16,616 smokers interviewed, smokers from urban areas accounted for a little more than half of the sample (8,502 subjects or 51.2%), and 8,114 (48.8%) subjects were from rural areas. This is consistent with the sixth Chinese Census data. Most of the sample had a high family income (≥¥30000) per year (62.0%), which is also consistent with the sixth Chinese Census data.

The sampling was carried out using a three-stage stratified sampling process. Firstly, all the areas were grouped into two categories (urban area and rural area). Secondly, probability sampling in urban areas was conducted by selecting primary sampling units (blocks); then a sample of the required number of households was selected in each block with 800 to 1,000 households. Thirdly, in rural areas, sampling was refined to the village level. Using map sampling method, a number of villages were randomly selected from the 36 counties. Then, a sample of the required number of households was selected according to the population size of the villages using probability sampling.

### 2.2. Data Collection and Quality Control

In the actual survey, eligible respondents were asked to complete a face-to-face interview with a standard questionnaire. The questionnaires were designed based on the existing literature and were translated into Chinese for the purpose of comparison and consistency with relevant studies. In households with more than one age-eligible person available, the subject unit was selected randomly. Each interview took about 10–15 minutes. The survey was conducted anonymously and the subjects could not be identified. All participants received a gift (RMB ¥5) as a monetary incentive for participation.

Strict quality control was taken during the whole process of sampling. All interviewers and supervisors were trained to ensure that the survey was consistent with the design, and a pilot study was carried out in one city before the actual survey. Some participants might see/hear the smoking bans/educational campaigns unconsciously. Although at the time of the survey, we just measured participants who claimed the smoking bans/educational campaign had a positive effect on them, when we checked the data accuracy and input the data, we only kept the data which showed consistency between subject's actual attitude and their verbal expression. All questionnaires were audited at each survey site to ensure 100% completion. Before data entry, 10% of them were reviewed by phone to ensure accuracy. All questionnaires were input by trained data entry personnel and data entry quality was assured by double data entry check. An experienced supervisor monitored the whole process of data entry to control the entry error rate under 1‰.

### 2.3. Measures

Intention to quit is the dependent variable in our study. Intention to quit was based on the question: “Do you plan to quit smoking?” Smokers who responded “within the next month,” “within the next 6 months,” or “sometime in the future after 6 months” were considered to have the intention to quit (coded as 1), whereas smokers who responded “not planning to quit” or “do not know” were considered to have no intention to quit (coded as 0). This measure was dichotomized for two reasons. First, our primary interest was to understand TC policy's effect on smoker's intention to quit, rather than by what time they were going to quit. Second, the frequency distribution of this measure was highly skewed with the majority being in the no intention category.

The factors driving the effectiveness level of the two policies (smoking bans and educational campaigns) are also explored in this study. The perceived effects of educational campaigns and smoking bans in public places were assessed by asking the question: “Do you think that educational campaigns (smoking bans in public places) have a positive effect on you?” Smokers who responded “reduce smoking,” “choose low-tar cigarettes,” or “choose low hazard cigarettes” were considered to be affected by the policies (coded as 1), whereas smokers who responded “smoke more,” “no effect,” or “do not know” were considered not to be affected (coded as 0).

The major independent variables in this study included subjects' demographic information, such as gender, population density, age, education, income, and number of cigarettes smoked per day. Age was categorized into three groups (18–29, 30–49, and 50+ years) and household income was divided into two groups (<¥30,000 and ≥¥30,000). Education was categorized into three groups (low: illiterate or having only primary school education; medium: high school or secondary school education in technique; and high: university or junior college education). Number of cigarettes smoked per day was categorized into three groups (1–10, 11–20, and 21+).

### 2.4. Statistical Analysis

Sample weights were simply calculated as the inverse 1/*p*(*i*) of its probability *p*(*i*) to be selected; the final weight for a sampled individual was the number of people in the city population and the sampling category represented by that individual [36]. SPSS for Windows version 17.0 (SPSS, Chicago, Illinois, USA) was used for all data analyses. Descriptive statistical analysis was used to examine the distribution of demographic characteristics in our sample. In the first analysis, we applied multivariate logistic regression models to examine the factors associated with the effects of educational campaigns and smoking bans in public places. Following the first study, we also employed the logistic regression models to determine to what extent intention to quit smoking was caused by educational campaigns and smoking bans in public places. All categorical variables were changed to dummy variables before entering the model. A level of *p* ≤ 0.05 is used to determine the level of statistical significance. For this research, only smokers were included in the analyses.

### 2.5. Ethics Statement

This work was approved by the Ethics Committee of University of Science and Technology of China. Trained interviewers clearly explained the significance, aims, and content of the survey to the potential participants at the beginning of each survey. The survey is household interviews, nonpublic, internal, and we promised to guarantee absolute privacy. The face-to-face interviews were then conducted if the participants signed informed consent. The participants could decide to withdraw from the interview anytime during the survey.

## 3. Results

Despite the perceived success of educational campaigns and smoking bans in public places in China, minimal rigorous analysis on their effects has been conducted. This study examines how the major characteristics of smokers affect the effectiveness of these two policies and whether these two policies can drive smokers to quit smoking. Such knowledge would have the potential to guide future TC policies and programs toward increasing the quitting rate in China. We hope our scientific work and results become a useful tool for advocating appropriate policy changes and will promote the FCTC implementation in China.

### 3.1. Effects of Smokers' Characteristics on the Effectiveness of Educational Campaigns and Smoking Bans

Table 1 shows the demographic characteristics of the survey sample. Of the 16,616 smokers interviewed, 16,098 are males (96.9%) and 518 are females (3.1%). The 30–49 years age group accounts for 56.2% of the sample. Most of the subjects have a low level of education (63%). Smokers from rural areas account for nearly half of the sample (48.8%). Most of the sample has a high family income (≥¥30000) per year (62.0%). Almost half of the smokers surveyed (48.6%) smoke 11–20 cigarettes per day.

Table 2 shows the results of a multivariate logistic regression that examines the factors associated with the effects of educational campaigns and smoking bans in public places. In the left section of educational campaigns, 49.5% of smokers thought that educational campaigns had a positive effect on themselves. The proportion of smokers affected by educational campaigns does not significantly differ across gender and household income. The difference between the 15–29 age group (reference group) and the 30–49 age group (OR = 1.12) is significant at the *p* ≤ 0.05 level. This may be because the 15–29 age group belongs to the youth group and the harm caused by smoking has not been recognized by them. While the 30–49 age group is mainly middle-aged smokers, they are more concerned about their health status; therefore, they are more sensitive to the knowledge involved in the educational campaigns. Comparing with smokers with a low education level, the effect of educational campaigns on those with either medium education level (OR = 1.20, 95% CI 1.11–1.30) or a high education level (OR = 1.56, 95% CI 1.14–1.72) is more significant. This shows that people with higher level of education are more knowledgeable; as a result, their understanding of the dangers of tobacco is more comprehensive, holding a more positive attitude toward educational campaigns; therefore, the greater likelihood is that they are affected. Smokers from urban areas are significantly less affected by educational campaigns (OR = 0.81) than those from rural areas. On the one hand, because rural residents are affected by the cultural level, the knowledge of the harm of tobacco is at a lower level. On the other hand, educational campaigns play a more important role for those who do not understand the dangers of smoking, so health educational campaigns in rural areas have a higher sensitivity. Smokers who tend to be heavy smokers are less likely affected by educational campaigns than light smokers.

The last three columns (right section) of Table 2 show the results of the logistic regression that examines the factors associated with the effects of smoking bans in public places. The figures show that 66.2% of cigarette consumers thought smoking bans in public places had a positive effect on themselves. The proportion of smokers between the 15–29 age group (reference group) and the 50+ age group (OR = 0.81) is significant at the *p* ≤ 0.05 level. Comparing with smokers from rural areas (reference group), smokers from urban areas are less likely affected by smoking bans in public places (OR = 0.82). This may be due to the fact that the current China's promotion strategy of tobacco control education is relatively simple; at the same time, smoking bans in public places are in their infancy, and urban areas need deeper and more targeted strategy. Smokers who tend to smoke a few cigarettes per day are less likely affected by smoking bans in public places. It shows that the greater the amount of cigarettes smoked, the greater the smokers' tobacco addiction, and the higher the degree of their dependence on nicotine, so that smokers are less likely to be influenced by other factors (smoking bans and educational campaigns). No significant difference is noted across gender, education, and household income. This survey results are somewhat to our surprise; to the factors of gender, education and household income, the possible reason is the current smoking bans in public places in Anhui Province are only advocate prescriptive, lacking appropriate executive body, and there are no specific penalties; thus, smoking in public places is a very common phenomenon. Therefore, its influence is relatively weak.

### 3.2. Effects of Educational Campaigns and Smoking Bans on Intention to Quit Smoking

Table 3 shows the results of the logistic regression that examines the factors associated with the intention to quit smoking. Smokers affected by educational campaigns and smoking bans in public places are more likely to have the intention to quit (OR = 2.58, 95% CI 2.27–2.94 and OR = 1.31, 95% CI 1.14–1.51, resp.). It indicates that both educational campaigns and smoking bans are effective in influencing smoker's behavior positively. It is not surprising that male smokers (OR = 0.74, 95% CI 0.57–0.97) have significantly less intention to quit smoking than female smokers; the social and culture factors can explain such pattern. Smokers from urban areas have significantly more intention to quit smoking (OR = 1.24) than smokers from rural areas (reference group). Relatively higher education and healthy lifestyle in urban areas can help smokers recognize the damage of smoking more comprehensively; as a result, they are more likely to quit smoking. Moreover, smokers who smoke 11–20 cigarettes per day have significantly less intention to quit smoking (OR = 0.80) than smokers who smoke 1–10 cigarettes per day (reference group). The level of addiction should be the cause behind the finding. Other factors associated with stronger intention to quit include medium and high education level and higher household income. The social class difference could lead to such result that high social class (high education and high household income) is more likely to quit smoking. However, the proportion of smokers with the intention to quit smoking is not significantly different for heavy smokers and age factors.

## 4. Discussion

The findings are representative and generalizable owing to the sampling design and the large sample size. Approximately half of the survey respondents thought that educational campaigns had a positive effect on themselves, and only one in three current smokers thought that smoking bans in public places had no effect. Smokers who were affected by the TC policies were associated with increased intention to quit smoking. Therefore, we conclude that these two TC approaches in general have positive effect in China. The combination of smoking bans and educational campaigns has the potential to build awareness of the harm of smoking and to support smoke-free legislation. Such effects will shape more favorable attitudes and social norms about quitting, which in turn can increase smokers' intention to quit [37]. Considering that smoking bans in public places and educational campaigns were widely ignored or not implemented into practice in China [11], China should spend more effort to accelerate the progress of TC legislation in public places and should implement various forms of public educational campaigns to increase knowledge of the health consequences of smoking.

Smokers who are from rural areas and smoking few cigarettes per day are more likely affected by these two policies. In China, the vast majority of the smoking population still live in rural areas, having a higher smoking prevalence [33, 38]. Currently, related educational campaigns and bans in rural areas are implemented at relatively less extent than urban areas. Therefore, our study suggests that more attention should be paid to rural areas when designing TC programs. In addition, the light smoker should be the focus as well since they may gradually become heavy smokers and they are sensitive to the policies. Therefore, by delivering message to them intensively, they have more chance of quitting smoke and prevent themselves from becoming heavy smokers. The research findings also suggest that policies should be tailored to the smoker's level of nicotine dependence; namely, heavier smokers should be treated with interventions to improve the effects of the TC policies. For that purpose, one option is to train doctors and health care professionals in providing brief cessation interventions or to implement a systematic cessation services referral program [39]. Another possible option is to provide a smoking cessation service hotline for mutual communication. Such actions can complement TC policies in achieving the expected objectives. Educational campaigns by mass media are effective in reaching large number of populations but are relatively expensive. Therefore, the appropriate media channel and frequency of airing can be chosen at targeted population economically [40]. In this study, individual-level factors, such as age, education level, population density, and number of cigarettes smoked per day, were found to be independently associated with the effects of educational campaigns. This finding suggests that the Chinese government and health authorities should take these factors into consideration when designing the content and forms of education activities correspondingly, so as to appeal to their target audiences more effectively. For example, smokers with higher education level are more affected by educational campaigns than those with low education level, implying the need to accommodate smokers' education level by implementing the educational campaigns differently.

Another key finding is the level of interest (overall 9.2%) in quitting among smokers from the 17 cities studied, which is lower than that reported by Feng et al. in six cities in China (ranging from 15% to 31%) with their survey limited to urban areas [33] and certainly considerably lower than four developed countries in the West countries ranging from 65% to 81% [41]. This finding emphasizes the need for a more dedicated effort to be made to encourage quitting among Chinese smokers, so that China can significantly reduce the health burden overall arising from tobacco-related diseases and death. Despite the low level of interest to quit smoke, factors such as gender, age, income, education, and nicotine dependence are associated with interest in quitting, which is consistent with the findings from Western countries [42, 43]. One thing that needs to be pointed out is that as mentioned in Table 2 smokers from urban areas were significantly less affected by educational campaigns, but as in Table 3 we conclude that smokers in urban areas had significantly more intention to quit. Urban residents have a faster pace of life and they are overloaded by the overwhelming information every day compared to rural residents, so they may be too busy to notice the two policies. However, those urban smokers aware of the two policies are more likely to quit smoke. This is consistent with the previous studies conducted in Asian region (e.g., Indian and South-Asian region) which suggested that tobacco users with lower sociogeographical status were associated with no intention to quit [44, 45]. Generally, urban residents tend to have higher income and higher level of education, they also have higher level of health awareness, and they may see/hear the educational campaigns unconsciously, but they better know the harm of smoking, which could cause higher level of intention to quit smoking. Research from Western countries also suggested that knowledge of the health effects of tobacco tended to be lower among population groups with lower socioeconomic status [46]. Some research showed that the knowledge of smoking-related illness and exposure to antismoking messages in newspaper, TV/radio, and educational campaigns could enhance the intention to quit smoking [45]. Thus, to those urban smokers who already knew these two policies, they had significantly more intention to quit.

Consistent with previous research in the effectiveness of educational campaigns in promoting quitting [12, 33, 47], we found the same factors in predicting intention to quit smoking. Due to the lack of awareness of the health risks among Chinese smokers [48], Chinese government should adopt all possible means to enhance its TC publicity and to build good social atmosphere of support for TC in the entire society and, at the same time, customize media and message to target smokers effectively and efficiently. This decisive approach may increase smokers' awareness of the adverse health effects of smoking and may be an effective way to increase the intention to quit smoking in China.

Moreover, this study identifies an association between the smoking bans in public places and increased intention to quit smoking. This result is consistent with the evidence from studies in the West [27, 49, 50] that the effectiveness of comprehensive smoke-free legislation (i.e., 100% smoke-free legislation, without exemptions or designated smoking rooms) in reducing per capita tobacco consumption existed. However, this is not the case for partial bans and restrictions. China still has only partial legislation, which significantly lowers its effect, especially in protecting nonsmokers from secondhand smoke. As per WHO recommendations, China should enforce the law in a more overwhelming manner, in the way of implementing comprehensive smoke-free legislation (i.e., ban smoking rooms, without exceptions), promoting local TC legislation, and running more educational campaigns.

## 5. Conclusions and Future Research

This study examines the effects of the two policies by major characteristics of smokers and whether the affected smokers have intention to quit smoking. Results show that smokers' individual-level factors such as age, education level, population density, and number of cigarettes smoked per day are associated with the effects of educational campaigns, while the effects of smoking bans in public places depend on individual-level factors such as age, population density, and number of cigarettes smoked per day. Interest in quitting smoke in China is influenced by factors, such as educational campaigns, smoking bans, gender, education level, population density, household income, and number of cigarettes smoked per day. Based on our findings, policy-makers should consider these factors when designing TC programs to ensure the effectiveness of their policy.

The advantages of this study include the large sample size, the rigorous sampling design, and a suitable approach to address the issue. In general, the expected findings are identified. However, this study also has some limitations. One limitation is the use of cross-sectional data, which limits our ability to explore causal relationships. Moreover, this study uses self-reported data that may be subject to social desirability and bias. Despite training the interviewers to be objective when conducting the survey they said training may not have completely solved the problem. Furthermore, social environmental factors, aside from demographic characteristics, may also be associated with the effects of the policy and smokers' intention to quit smoking. These factors will be examined in future studies. Although the sample is a good representative of the whole population and the results can be generalized to provide a general guidance in China, the analysis can be done in other areas to validate the robustness of our findings.

## Figures and Tables

**Table 1:** Demographic characteristics of the survey sample.

Characteristics	*N*	Percentage	Characteristics	*N*	Percentage
Gender			Population density		
Female	518	3.1	Rural	8114	48.8
Male	16098	96.9	Urban	8502	51.2
Age			Household income (¥)		
15–29	3106	18.7	<30000	4733	28.5
30–49	9338	56.2	≥30000	10301	62.0
50+	4172	25.1	Not available	1582	9.5
Education			Number of cigarettes smoked per day		
Low	10467	63.0	1–10	5131	30.9
Medium	3817	23.0	11–20	8076	48.6
High	2332	14.0	21+	3409	20.5

**Table 2:** Result of logistic regression examining factors associated with the effects of educational campaigns and smoking bans in public places.

	Percentage affected by educational campaigns	OR	95% CI	*p* value	Percentage affected by smoking bans	OR	95% CI	*p* value
Gender								
Female	52.7	Reference			65.3	Reference		
Male	49.4	0.96	0.81–1.15	0.687	66.2	1.04	0.86–1.25	0.718
Age								
15–29	52.1	Reference			68.5	Reference		
30–49	50.2	**1.12**	1.03–1.22	0.011	67.2	0.98	0.89–1.07	0.676
50+	46.1	0.99	0.89–1.10	0.820	62.3	**0.81**	0.73–0.90	0.000
Education								
Primary school	46.9	Reference			65.8	Reference		
Secondary school	51.3	**1.20**	1.11–1.30	0.000	65.7	0.98	0.90–1.07	0.680
High school or higher	58.3	**1.56**	1.41–1.72	0.000	68.6	1.10	0.99–1.23	0.084
population density								
Rural	50.9	Reference			67.9	Reference		
Urban	48.2	**0.81**	0.76–0.87	0.000	64.5	**0.82**	0.77–0.88	0.000
Household income (¥)								
<30000	49.1	Reference			65.3	Reference		
≥30000	49.8	0.97	0.90–1.05	0.438	66.8	1.05	0.97–1.13	0.267
Cannot say	48.9	0.92	0.82–1.04	0.183	65.1	0.95	0.84–1.08	0.429
Number of cigarettes smoked per day								
1–10	57.8	Reference			68.3	Reference		
11–20	47.7	**0.69**	0.64–0.74	0.000	66.2	**0.93**	0.86–1.00	0.000
21+	41.4	**0.54**	0.50–0.60	0.000	63.2	**0.82**	0.75–0.91	0.000

^∗^Bold values indicate a significant difference at *p* ≤ 0.05.

**Table 3:** Results of logistic regression examining factors associated with any intention to quit smoking.

	Percentage intending to quit	OR	95% CI	*p* value
Gender				
Female	13.5	Reference		
Male	9.1	**0.74**	0.57–0.97	0.029
Age				
15–29	10.4	Reference		
30–49	9.3	1.05	0.91–1.21	0.524
50+	8.2	1.02	0.86–1.22	0.792
Education				
Primary school	7.9	Reference		
Secondary school	10	**1.15**	1.00–1.32	0.045
High school or higher	13.9	**1.50**	1.28–1.76	0.000
Locality type				
Rural	8	Reference		
Urban	9.1	**1.24**	1.10–1.39	0.000
Household income (¥)				
<30000	9.1	Reference		
≥30000	9.2	**0.82**	0.72–0.94	0.003
Cannot say	9.5	0.90	0.74–1.10	0.311
Number of cigarettes smoked per day				
1–10	11.7	Reference		
11–20	8.1	**0.80**	0.70–0.90	0.000
21+	8.2	0.91	0.77–1.07	0.235
Educational campaigns				
No	5	Reference		
Yes	13.5	**2.58**	2.27–2.94	0.000
Smoking bans				
No	5.8	Reference		
Yes	10.9	**1.31**	1.14–1.51	0.000

^∗^Bold values indicate a significant difference at *p* ≤ 0.05.
